# Signal Transmission in a Human Body Medium-Based Body Sensor Network Using a Mach-Zehnder Electro-Optical Sensor

**DOI:** 10.3390/s121216557

**Published:** 2012-11-30

**Authors:** Yong Song, Qun Hao, Kai Zhang, Jingwen Wang, Xuefeng Jin, He Sun

**Affiliations:** School of Opto-Electronic, Beijing Institute of Technology, Beijing 100081, China; E-Mails: yongsong@bit.edu.cn (Y.S.); 20040491@bit.edu.cn (K.Z.); kedawangjingwen@163.com (J.W.); imllbh@gmail.com (X.J.); sunhe_bit@sina.cn (H.S.)

**Keywords:** BSN, IBC, electro-optical sensor, signal transmission, human body

## Abstract

The signal transmission technology based on the human body medium offers significant advantages in Body Sensor Networks (BSNs) used for healthcare and the other related fields. In previous works we have proposed a novel signal transmission method based on the human body medium using a Mach-Zehnder electro-optical (EO) sensor. In this paper, we present a signal transmission system based on the proposed method, which consists of a transmitter, a Mach-Zehnder EO sensor and a corresponding receiving circuit. Meanwhile, in order to verify the frequency response properties and determine the suitable parameters of the developed system, *in-vivo* measurements have been implemented under conditions of different carrier frequencies, baseband frequencies and signal transmission paths. Results indicate that the proposed system will help to achieve reliable and high speed signal transmission of BSN based on the human body medium.

## 1. Introduction

Signal transmission based on the human body, also termed intra-body communication (IBC), is a technology using the human body as the transmission medium for electrical signals [1]. Compared with short distance wireless communication technologies, such as Bluetooth and Zigbee, *etc*., this technology has several novel characteristics, which can be summarized as follows: (1) due to the fact that the signal mainly transmits within the human body and little radiation leaks out, it avoids the disturbance of environmental electromagnetic noise and can achieve comparatively higher data rates; (2) as a special type of cable communication using the human body as transmission medium, it needs comparatively lower energy consumption [2]; (3) using this technology, communication can be started or stopped by touching, standing and sitting down of the human body [3]. Due to the advantages mentioned above, it is believed that the signal transmission technology based on the human body medium will offer significant advantages in BSNs used for healthcare [1,4] and other related fields.

Sensors used for signal detection are very important for achieving reliable signal transmission based on the human body. Firstly, to guarantee the safety of the human body, signals injected into the human body should be low [1,5]. Moreover, the impendence of human body also results in signal attenuation [6], therefore, sensors used for detecting the signal transmission within the human body should have high sensitivity. Secondly, with the influence from the floating ground of wearable electronical devices, signal transmission within the human body may suffer from great distortion, therefore, anti-interference properties are another important characteristic required by the sensor. Recently, two kinds of sensors have been chosen, the electrical sensor and electro-optical (EO) sensor. However, due to the fact that the electrical sensor has comparatively low input impedance and is easy to be interfered with by electromagnetic noise, the typical signal transmission distance based on this kind of sensor is only approximately 30 cm and the corresponding signal transmission rate is limited to 40 kbps [3]. As for the EO sensor, due to its extremely high input impedance, the influence of the electrical noise can be greatly decreased. Moreover, the ground electrode of the EO sensor is electrically isolated from the electronic circuits, which eliminates the influence of the floating ground potential [7]. As a result, both the noise and the distortion of the receiving signal can be greatly decreased, thereby high signal transmission rate can be achieved [3]. Therefore, EO sensors are believed to be suitable for detecting signal transmissions within the human body.

On the other hand, recent works with respect to the EO sensor used for signal transmission based on the human body medium mainly focus on sensors based on bulk electro-optical crystals [3,7,8]. This kind of EO sensor has an additional phase delay caused by natural binary refraction, which is very sensitive to the temperature that thereby influences the signal transmission quality. Moreover, the phase delay of this kind of EO sensor depends on the aspect ratio of EO crystals, which results in the comparatively big size of the EO sensor and will limit its application in BSNs. In our previous works, we proposed a novel signal transmission method based on the human body medium by using a Mach-Zehnder EO sensor, which will help to achieve signal transmission based on the human medium with the characteristics of good temperature dependence properties, small size and low power consumption [9]. In this paper, we present a signal transmission system based on the proposed method, and the frequency response properties as well as the parameters of the proposed system have been discussed. Firstly, we described the proposed signal transmission system, which consists of a transmitter, a Mach-Zehnder EO sensor and a corresponding receiving circuit. Secondly, the advantage with respect to the frequency response of the signal transmission based on the human body medium by using the proposed system has been verified by using *in-vivo* measurements. Furthermore, in order to determine the suitable parameters, the corresponding *in-vivo* signal transmission experiments with different carrier frequencies, baseband frequencies and multi-paths were implemented. Results indicate that the proposed method will help to achieve reliable and high speed signal BSN transmissions for healthcare and the other related fields. The rest of the paper is organized as follows. Section 2 describes the signal transmission system based on human body medium. Section 3 mainly focuses on the experiments and results discussion. Section 4 concludes the paper.

## 2. Signal Transmission System

Generally, the signal transmission approaches based on the human body medium can be divided into two types, which include electrostatic coupling type and galvanic coupling type [6,10]. Compared with the latter type, the former has the characteristic of less signal attenuation, which is very important for decreasing power consumption. Therefore, in our investigation the electrostatic coupling type was chosen as the approach for signal transmission based on the human body medium.

### 2.1. System Structure

**Figure 1.**
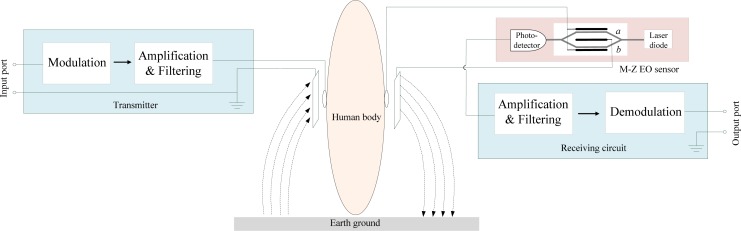
The developed signal transmission system based on the human body medium.

The developed signal transmission system based on the human body medium is composed of a transmitter, a Mach-Zehnder EO sensor and a receiving circuit, as shown in Figure 1. In the developed system, a baseband signal is input to the transmitter through the input port first, and then it is modulated and amplified in the transmitter. The processed signal is coupled into the human body through the signal electrode, while it is also coupled into the earth ground through the ground electrode. Subsequently, signal transmission within the human body is received by a Mach-Zehnder EO sensor through the signal electrode. In the EO sensor, signal is coupled to the ground electrode of the Mach-Zehnder modulator, and then it transmits to the ground electrode of the receiver. Finally, signal is coupled into the earth ground, and thereby a signal loop has been established.

On the other hand, the functions of the Mach-Zehnder electro-optical modulator can be described as follows: as shown in Figure 1, the Mach-Zehnder EO sensor consists of a laser diode, a Mach-Zehnder EO modulator and a photodetector. As the laser light (λ = 1,550 nm) from the laser diode passes through the two arms of the Mach-Zehnder EO modulator, the refractive index of the arm changes with the voltage of the received signal which is applied on it. Meanwhile, the modulator sums the optical waves from each arm, and thereby the phase change is converted into amplitude change. Subsequently, the change of optical amplitude is converted into the corresponding change of electric signals by the photodetector. Finally, the output signal of the photodetector is processed by the receiving circuit. As shown in Figure 1, the ground electrode is insulated from the photodetector and the receiving circuit, which will help to decrease signal noise and waveform distortion.

### 2.2. Transmitter

**Figure 2.**
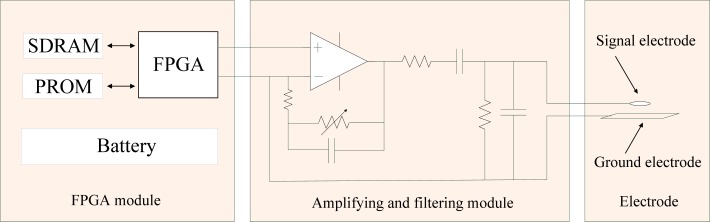
The transmitter structure.

In the developed signal transmission system, the transmitter is mainly used for modulating the baseband signal and coupling it into the human body. As shown in Figure 2, the transmitter can be divided into a Field Programmable Gate Array (FPGA) module, amplifying and filtering module, and electrostatic coupling electrode. Additionally, the baseband signal is stored in the FPGA module, thereby no input port is integrated in the developed transmitter. The structure and function of the three modules can be described as follows: the FPGA module consists of a FPGA (ALTERA EP1C6, EP3C40), a Synchronous Dynamic Random Access Memory (SDRAM), a Programmable Read Only Memory (PROM) and a battery, *etc*. Firstly, it generates a carrier signal with the required frequency, then the carrier signal is modulated by the baseband signal which is stored in the PROM (16 Mbit) with the Differential Binary Phase Shift Keying (DBPSK) method. The amplifying and filtering module consists of a voltage amplifier and a band-pass filter. Due to the fact the Mach-Zehnder electro-optic modulator has extremely high input impedance, therefore, rather than using a complex constant current source to provide enough receiving signal amplitude in the transmitter based on electric sensor [11], here we can use a simple voltage amplifier with adjustable gain for amplifying the modulated signal. Additionally, a 500 kHz–30 MHz band-pass filter is added for filtering out the useless harmonic noise. Finally, the processed signal is coupled into human body by a customized copper electrode, which consists of a circular signal electrode with a radius of 10 mm and a rectangular ground electrode with a size of 10 cm × 2 cm. The signal electrode is parallel to the ground electrode, while the distance between them is 10 cm. Meanwhile, the signal electrode is connected with the ground electrode by using rubber material, which has a relative permittivity of 2.6.

### 2.3. Mach-Zehnder EO Sensor

**Figure 3.**
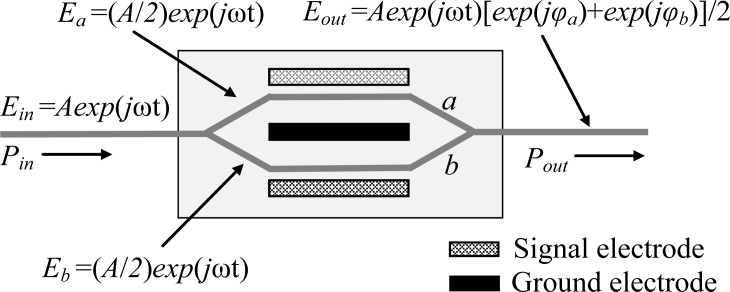
The principle of Mach-Zehnder EO sensor.

As shown in Figure 1, our Mach-Zehnder EO sensor was composed of a laser diode, a Mach-Zehnder EO modulator and a photodetector. The principle of the Mach-Zehnder EO sensor can be described as follows: as shown in Figure 3, supposing that the electric field of the incident input light *E_in_* = *Aexp*(*j*ωt), the electric fields in the two arms (*E_a_* and *E_b_*) of the Mach-Zehnd EO modulator can be described as *E_a_* = *E_b_* = (*A/2*)*exp*(*j*ωt). Therefore, the electric field corresponding to the emergent light of the Mach-Zehnder EO modulator (*E_out_*) can be expressed as [12]:

(1) $$\begin{array}{l} E_{\mathrm{out}}=\frac{A}{2}\text{exp}(j\omega t)[\text{exp}(j\varphi_{a})+\text{exp}(j\varphi_{b})]\\ \;\;\;\;\;\;\;\;\;=A\text{exp}\{j[\omega t+\frac{2\pi l}{\lambda_{0}}(n_{e}-n_{o})]\}\cdot \text{cos}[\frac{\Gamma \pi L}{G\lambda_{0}}(n_{e}^{3}\gamma_{33}-n_{o}^{3}\gamma_{13})V_{e}+\varphi_{0}], \end{array}$$

where *φ_a_* and *φ_b_* represent the phases of the arm *a* and *b*, respectively, *l* is the length of the EO crystal, *λ*_0_ is the optical wavelength, *n_e_* and *n_o_* are the extraordinary refractive index and ordinary refractive index of EO crystal, respectively, *Γ* is the overlap integral factor representing the interaction between the electric field applied on the electrodes and the light wave field, *L* is the electrode length, *G* is the distance between the signal electrode and the ground electrode of the Mach-Zehnder EO modulator, *γ*_33_ and *γ*_13_ are the electro-optic coefficients of the EO crystal, *V_e_* represents the voltage applied on the EO crystal which represents the signal voltage transmitting within the human body, and *φ*_0_ is the phase difference used for setting the operating point of the Mach-Zehnder EO modulator.

Subsequently, the relationship between the input optical power (*P_in_*) and the output optical poer (*P_out_*) of the Mach-Zehnder EO modulator can be expressed as:

(2) $$P_{\mathrm{out}}=P_{\mathrm{in}}{\text{cos}}^{2}[\frac{\Gamma \pi L}{G\lambda_{0}}(n_{e}^{3}\gamma_{33}-n_{o}^{3}\gamma_{13})V_{e}+\varphi_{0}].$$

Finally, the output voltage of the photodetector (*V_out_*), which represents the output voltage of the whole Mach-Zehnder EO sensor, can be expressed as [13]:

(3) $$V_{\mathrm{out}}=10^{-10/k}SR_{k}P_{\mathrm{out}}=10^{-10/k}SR_{k}P_{\mathrm{in}}{\text{cos}}^{2}[\frac{\Gamma \pi L}{G\lambda_{0}}(n_{e}^{3}\gamma_{33}-n_{o}^{3}\gamma_{13})V_{e}+\varphi_{0}].$$

where *k* is the insertion loss of the modulator, and *S* and *R_k_* represent the conversion efficiency and the transimpedance of the photodetector, respectively. Therefore, the receiving signal voltage (*V_e_*) transmitting within the human body can be achieved by measuring the output voltage of the photodetector (*V_out_*).

### 2.4. Receiving Circuit

**Figure 4.**
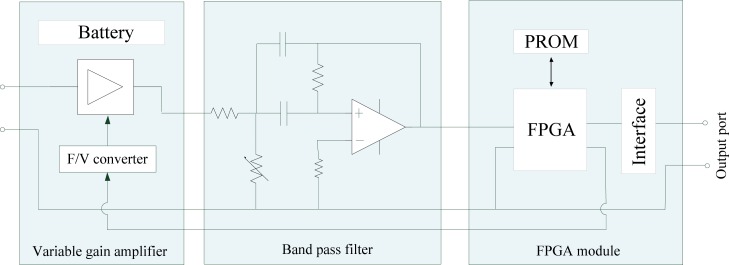
The structure of receiving circuit.

The receiving circuit is mainly used for amplifying, filtering and demodulating the signal output from the Mach-Zehnder EO sensor. As shown in Figure 4, our receiving circuit consists of a variable gain amplifier, a band-pass filter, a FPGA module and a battery, *etc*. In order to provide a signal with appropriate amplitude for the demodulation module based on FPGA, a variable gain voltage amplifier with functions of coarse adjustment and accurate adjustment was developed, in which the coarse adjustment (linear gain control) can be used for controlling gain artificially, while the accurate adjustment (index gain control) makes it possible to control the output signal voltage automatically according to the signal from FPGA through a Frequency to Voltage (F/V) converter circuit. Moreover, an active band-pass filter was developed for filtering noises. Finally, the receiving modulated signal is demodulated by the FPGA module, and thereby the original baseband signal can be achieved at the output interface of the receiving circuit.

## 3. Experiments and Discussion

### 3.1. Frequency Response

The frequency response of the signal transmission system based on the human body medium is an important factor that influences the quality of the receiving signal. In our investigation, the frequency response of the signal transmission based on the proposed Mach-Zehnder EO sensor was determined, while the corresponding *in-vivo* measurements with respect to the signal transmission based on electronic sensor was also carried out under the same conditions for comparison.

#### 3.1.1. Method

**Figure 5.**
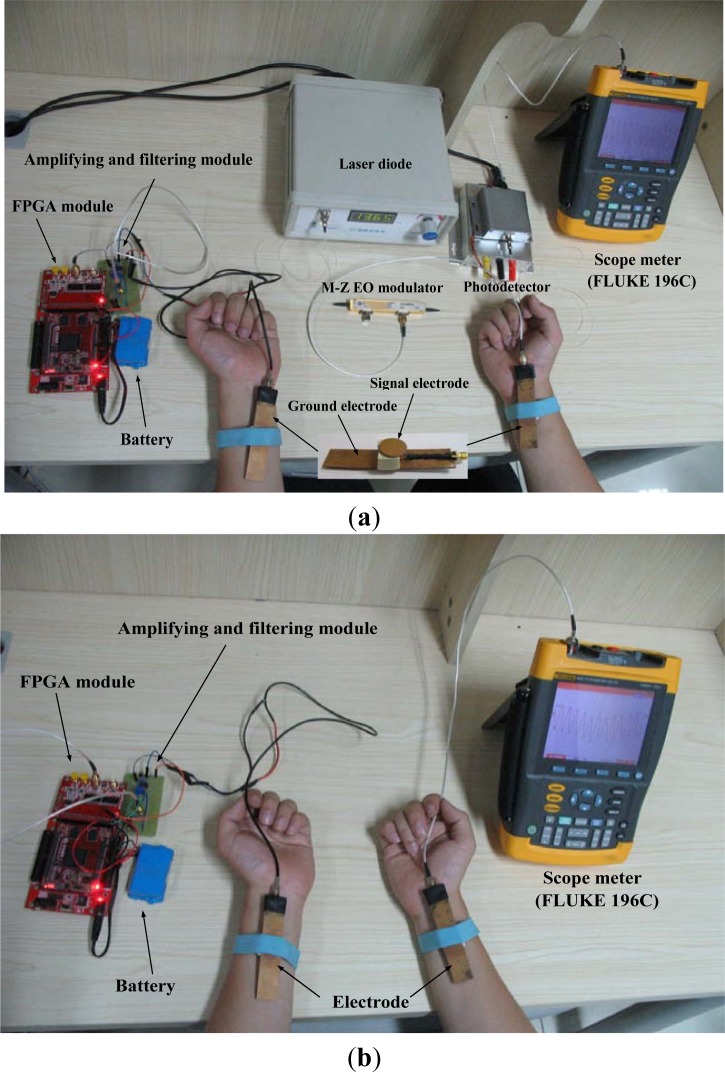
Experiment device with respect to the frequency response of signal transmission system based on human body, in which (**a**) is the experiment device based on Mach-Zehnder EO sensor, and (**b**) is the experiment device based on electronic sensor.

Our experimental device, as shown in Figure 5(a), includes a transmitter, a proposed sensor which consists of a laser diode, a Mach-Zehnder EO modulator (10 Gb/s intensity modulator, made by JDS Uniphase Corporation, Milpitas, CA, USA) and a photodetector. Moreover, a scope meter (FLUKE 196C) was used for displaying the receiving signal. Additionally, to simulate the actual application of BSN, the transmitter and the scope meter were powered by a battery module. On the other hand, a 27 year-old male with 80 kg weight and 182 cm height was chosen as the subject. Figure 5(b) shows the experiment device with respect to the frequency response of the signal transmission system based on electronic sensor, in which the same transmitter shown in Figure 5(a) was used, while the scope meter serves as an electronic sensor.

#### 3.1.2. Results and Discussion

**Figure 6.**
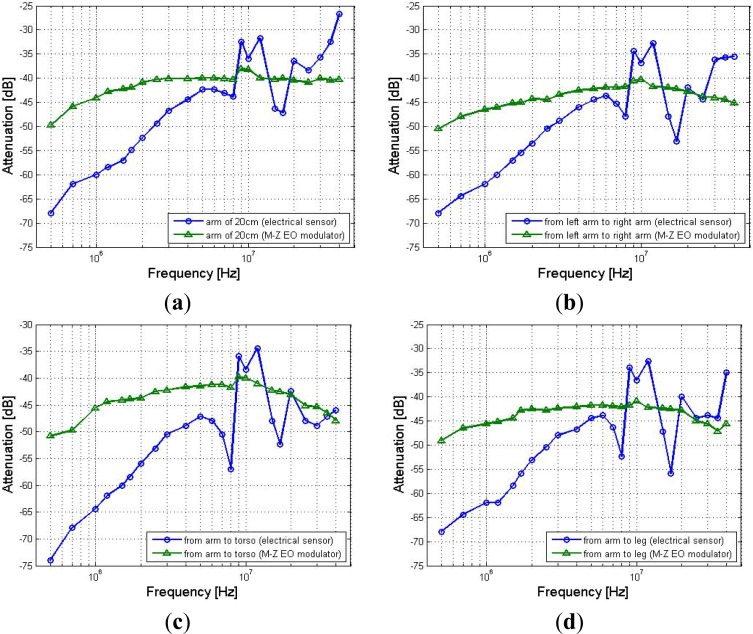
The experiment results of the frequency response corresponding to the signal transmission paths of (**a**) arm (20 cm), (**b**) left arm-right arm, (**c**) torso-arm and (**d**) leg-arm.

Figure 6 shows the *in-vivo* measurement results with respect to the frequency response of the signal transmission system based on the proposed EO sensor and the electronic sensor, respectively. It can be found from Figure 6 that signal frequency within the 2 MHz–30 MHz range has comparatively less effect on the signal attenuation in the signal transmission based on the Mach-Zehnder EO sensor along the different signal transmission paths, which include arm (20 cm), left arm-right arm (120 cm), torso-arm (70 cm) and leg-arm (180 cm). In contrast, it has comparatively greater effect on the signal attenuations of the signal transmission based on an electronic sensor. As shown in Figure 6(a), the results corresponding to the (20 cm) arm path with respect to the Mach-Zehnder EO sensor almost remain invariant within the frequency range of 2 MHz–30 MHz, while the maximum deviation of the results is only 2.85 dB. However, the corresponding results with respect to the electronic sensor have comparatively greater variation, while the maximum deviation is up to 20.70 dB. Additionally, even though both the signal attenuation curves shown in Figure 6(a) decrease gradually with the increase of signal frequency from 500 kHz to 2 MHz, the results referring to the Mach-Zehnder EO sensor are far less than the corresponding results referring to the electronic sensor. On the other hand, as shown in Figure 6(b,c,d), a similar phenomenon can also be found from the results corresponding to the paths of arm-right arm, torso-arm and leg-arm, which also indicate that the results referring to the Mach-Zehnder EO sensor have less variance in the frequency range of 2 MHz–30 MHz, while the results referring to the electronic sensor have comparatively greater variation. This phenomenon indicates that compared with the electrical sensor-based signal transmission, the proposed Mach-Zehnder EO sensor has a comparatively steady frequency response in the frequency range of 2 MHz–30 MHz. Meanwhile, the above phenomenon can be explained by the influences of the extremely high input impedance of the EO sensor as well as the electrically isolation between the ground electrode of the EO sensor and the receiving circuit [7,9]. Finally, if the signal frequency or the carrier frequency is set in this range, high quality of the signal transmission based on the human body medium will be expected.

### 3.2. Signal Transmission of BSN

#### 3.2.1. Method

**Figure 7.**
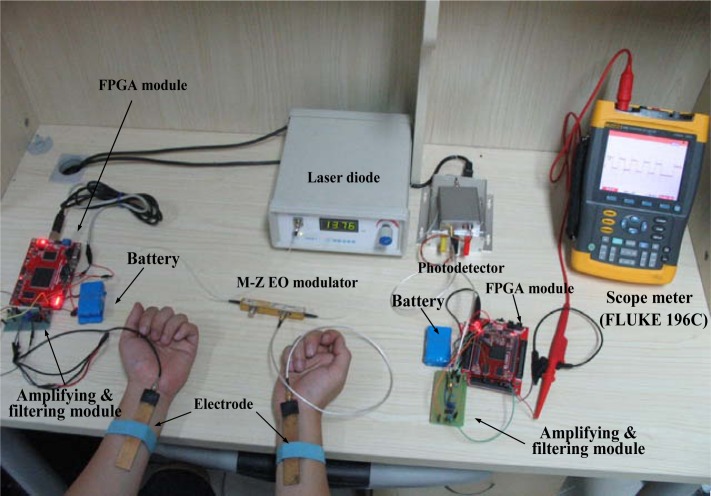
Experiment device used for the signal transmission experiments of BSN based on human body medium.

In order to verify the functions and determine the suitable parameters of the proposed signal transmission system used in the BSN, the corresponding *in-vivo* experiments with different carrier wave frequencies, baseband frequencies and multi-paths were implemented using the experimental device shown in Figure 7. Compared with the experimental device shown in Figure 5(a), a receiving circuit mentioned in Section 2.4, which includes a variable gain amplifier, a band pass filter and a FPGA module, was added to the experimental device. In our experiments, the signal “1010101010” was chosen as the baseband signal. In the FPGA module of the transmitter, the baseband signal was converted into the corresponding relocatable code singal (110011001100) first, and then it modulated the carrier wave signal with the DBPSK method. Subsequently, the modulated signal was coupled into the human body at the transmitting terminal and received by the proposed Mach-Zehnder EO sensor at the receiving terminal. Finally, the signal was processed and demodulated by the receiving circuit.

#### 3.2.2. Influence of Carrier Frequency

The experiments mainly focus on the influence of carrier frequency on the signal transmission based on the human body medium, in which 500 kHz was chosen as the baseband frequency (BF), while the carrier frequency (CF) was set as 1, 4, 8 and 16 MHz, respectively. As shown in Figure 7, the signal was transmitted from the left arm and received at the right arm.

**Figure 8.**
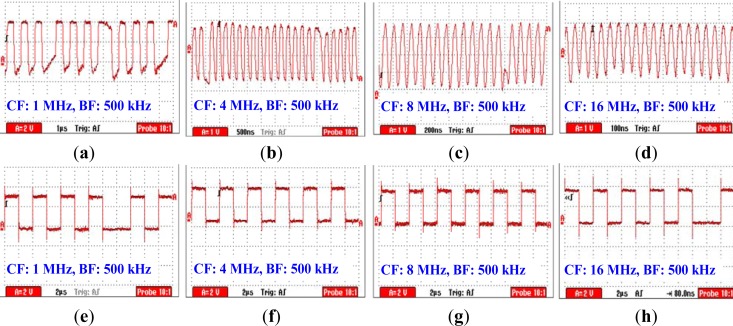
The modulated signal received by the Mach-Zehnder EO sensor on conditions that BF = 500 kHz and CF is (**a**) 1 MHz, (**b**) 4 MHz, (**c**) 8 MHz and (**d**) 16 MHz, respectively. Figure (**e**,**f**,**g**,**h**) are the corresponding demodulated signals of the modulated signals shown in Figure (**a**,**b**,**c**,**d**), respectively.

Figure 8(a) shows the modulated signal received by the Mach-Zehnder EO sensor under the condition that the CF = 1 MHz. We can find from Figure 8(a) that the modulated signal has distortion, especially at the junction between the signals representing “0” and “1”. Meanwhile, error codes can be found in the corresponding demodulated signal shown in Figure 8(e), while the measurement result of bit error rate (BER) is 1.25%. Furthermore, when the CF = 4 and 8 MHz, the quality of the modulated signal was increased, as shown in Figure 8(b,c). As a result, less error code has been found in the corresponding demodulated signal shown in Figure 8(f,g), while the measurement results of BER are 0.78% and 0.56%, respectively. On the other hand, the amplitudes of the demodulated signal shown in Figure 8(d) corresponding to CF = 16 MHz are decreased, and error codes can be found in the corresponding demodulated signal shown in Figure 8(h). Moreover, the BER measurement result is up to 1.62%. The phenomenon mentioned above can be explained as follows: (1) due to the fact that signal with the frequency less than 2 MHz generally results in comparatively high signal attenuation according to the results shown in Figure 6, thereby leading to the increasing of BER; (2) compared with the other three modulated signals shown in Figure 8, the modulated signal corresponding to CF = 16 MHz has more high frequency spectral. Due to the fact that signal attenuation also increases when signal frequency is higher than 20 MHz, thereby this results in the increase of the measured BER corresponding to CF = 16 MHz. Finally, as a conclusion, the carrier frequency used in BSN based on the human body medium should be higher than 1 MHz, while 8 MHz is a suitable frequency for CF.

#### 3.2.3. Influence of Baseband Frequency

The experiments with respect to the influence of baseband frequency over the signal transmission system based on the human body medium has been implemented using the experimental device shown in Figure 7. Meanwhile, the carrier frequency was set as 8 MHz, and the baseband frequency was set as 100 kHz, 1 MHz, 4 MHz and 8 MHz, respectively.

**Figure 9.**
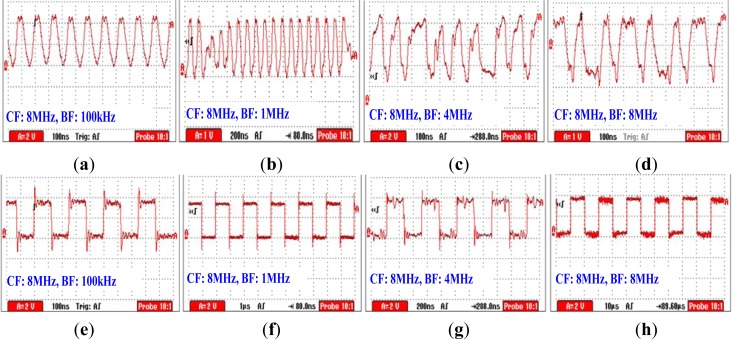
The modulated signal received by the Mach-Zehnder EO sensor on the conditions that CF = 8 MHz and BF is (**a**) 100 kHz, (**b**) 1 MHz, (**c**) 4 MHz and (**d**) 8 MHz, respectively. Figure (**e**,**f**,**g**,**h**) are the corresponding demodulated signals of the modulated signal shown in Figure (**a**,**b**,**c**,**d**), respectively.

Figure 9(a) shows the modulated signal measured by the proposed EO sensor under the condition that BF = 100 kHz, while Figure 9(e) shows the corresponding demodulated signal processed by the receiving circuit. We can find from Figure 9(a,e) that both the modulation and the demodulation can be implemented correctly in this case, while the measured BER is 0.59%. Meanwhile, Figure 9(b) shows the modulated signal under the condition that BF = 1 MHz, while Figure 9(f) is the corresponding demodulation signal. we can find from Figure 9(b,f) that even the pulses corresponding to the conversion process (from “1” to “0” or from “0” to “1”) has a comparatively smaller amplitude, the correct demodulation signal can also be achieved by using the receiving circuit integrated with variable gain amplifier, while the measured BER is 0.49%. Moreover, as shown in Figure 9(c,d), when BF was set as 4 MHz and 8 MHz, the pulses corresponding to the conversion process have almost the same amplitudes as the pulses representing “1” or “0”. Additionally, even some wave distortions can be found from the modulated signals shown in Figure 9(c,d), these distortions were limited in an acceptable range and thereby results in the correct demodulation signals shown in Figure 9(g,h). As a result, the measured BER’s corresponding to BF = 4 and 8 MHz are 0.68% and 0.73%, respectively.

#### 3.2.4. Influence of Signal Transmission Path

**Figure 10.**
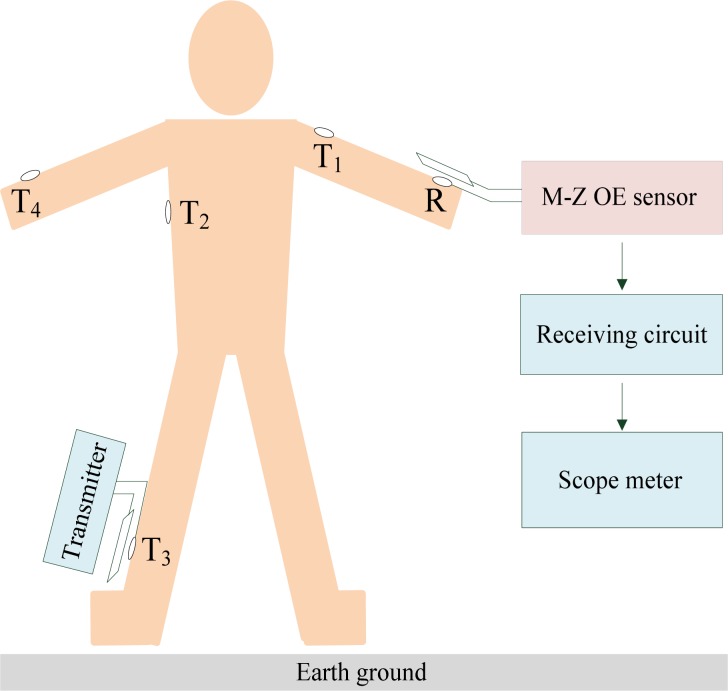
The experiment referring to the influence of signal transmission path in BSN based on human body medium, while the signal transmission paths include the arm path (*T_1_*-*R*), torso-arm (*T_2_*-*R*), leg-arm (*T_3_*-*R*) and left arm-right arm (*T_4_*-*R*).

In the signal transmission system based on the human body medium used in a BSN for healthcare, generally, biomedical sensors and the corresponding transmitters are located at different positions on the human body, and send their data to a sink node located somewhere on the human body (such as the wrist). Therefore, the influence of the signal transmission path within the human body should be considered. As shown in Figure 10, the receiving electrode was fixed on the position *R* of the human body, while the transmitting electrode was fixed on the positions *T_1_*, *T_2_*, *T_3_* and *T_4_*, respectively, thereby resulting in signal transmission paths with different lengths, which include the arm path (*T_1_*-*R*, 20 cm), torso-arm (*T_2_*-*R*, 70 cm), leg-arm (*T_3_*-*R*, 180 cm) and left arm-right arm (*T_4_*-*R*, 120 cm) in our experiment.

**Figure 11.**
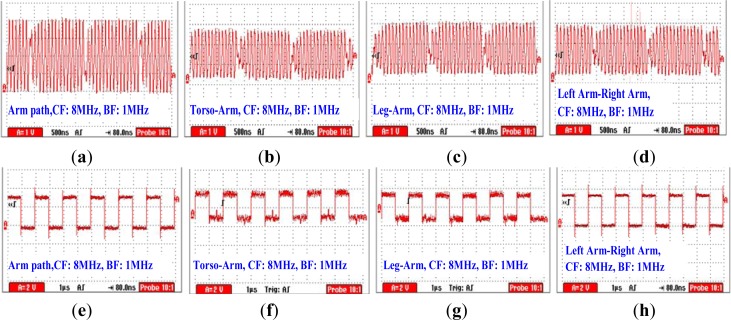
The modulated signal and the corresponding demodulated signal with respect to the four signal transmission paths (arm path, torso-arm, leg-arm, left arm-right arm) on the conditions that CF = 8 MHz, BF = 1 MHz.

Figure 11(a–d) show the modulated signals corresponding to the four signal transmission paths (*T_1_*-*R*, *T_2_*-*R*, *T_3_*-*R* and *T_4_*-*R*) measured by the proposed EO sensor under the conditions that CF = 8 MHz, BF = 1 MHz, while Figure 11(e,f,g,h) are the demodulated signals corresponding to Figure 11(a,b,c,d), respectively. According to Figure 11(a), we can find that the modulated signal has comparatively bigger amplitude. This phenomenon can be explained by the fact that signal transmission within the arm has less attenuation compared with the other three paths. Generally, a shorter length of the signal transmission path will result in a smaller signal attenuation [14,15]. Moreover, the comparatively high quality of the demodulated signal can also be achieved in this case, as shown in Figure 11(e). As a result, the measured BER is only 0.32% in this case. On the other hand, it can be found from Figure 11(b,c,d) that the modulated signals corresponding to the paths of torso-arm, leg-arm and left arm-right arm have similar waveforms and amplitudes. Meanwhile, according to the corresponding demodulated signals shown in Figure 11(f,g,h), all the signals shown in Figure 11(b,c,d) can be demodulated correctly, and the measured BER’s corresponding to Figure 11(f,g,h) are 0.47%, 0.62% and 0.49%, respectively. Therefore, it can be deduced that signal transmission path generally has less influence on the quality of the signal transmission based on the human body medium.

## 4. Conclusions

In this paper, a BSN signal transmission system based on the human body medium using a Mach-Zehnder EO sensor has been proposed. We demonstrated that, compared with the signal transmission system based on an electrical sensor, the proposed system based on a Mach-Zehnder OE sensor has a steady frequency response in the frequency range of 2 MHz–30 MHz. Furthermore, the corresponding *in-vivo* signal transmission experiments with different carrier wave frequencies, baseband frequencies and multi-paths were implemented, and some conclusions can be drawn as follows: (1) generally, the carrier frequency used in BSN based on the human body medium should be higher than 1 MHz, while 8 MHz is a suitable frequency; (2) using the proposed method, signal transmission with the BF range of 100 kHz–8 MHz can be achieved under the condition of CF = 8 MHz; (3) the signal transmission path has less influence to the quality of the discussed signal transmission based on the human body medim (electrostatic coupling type). Our results indicate that the proposed system will help to achieve reliable and high speed signal transmission based on the human body medium in BSNs used for healthcare and other related fields.

## References

[1] Zimmerman T.G. (1995). Personal Area Networks (PAN): Near-Field Intra-Body Communication.

[2] Wegmueller M.S., Oberle M., Felber N., Kuster N., Fichtner W. (2010). Signal Transmission by Galvanic Coupling Through the Human Body. IEEE Trans. Instrum. Meas.

[3] Shinagawa M., Fukumoto M., Ochiai K., Kyuragi H. (2004). A Near-Field Sensing Transceiver for Intrabody Communication Based on the Electrooptic Effect. IEEE Trans. Instrum. Meas.

[4] Wegmueller M.S., Huclova S., Froehlich J., Oberle M., Felber N., Kuster N., Fichtner W. (2009). Galvanic Coupling Enabling Wireless Implant Communications. IEEE Trans. Instrum. Meas.

[5] Hachisuka K., Nakata A., Takeda T., Shiba K., Sasaki K., Hosaka H., Itao K. (2003). Development of Wearable Intra-Body Communication Devices. Sens. Actuators A.

[6] Wegmueller M.S., Kuhn A., Froehlich J., Oberle M., Felber N., Kuster N., Fichtner W. (2007). An Attempt to Model the Human Body as a Communication Channel. IEEE Trans. Biomed. Eng.

[7] Sasaki A., Shinagawa M., Ochiai K. (2009). Principles and Demonstration of Intrabody Communication with a Sensitive Electrooptic Sensor. IEEE Trans. Instrum. Meas.

[8] Sasaki A., Shinagawa M. (2008). Principle and Application of a Sensitive Handy Electrooptic Probe for Sub-100-MHz Frequency Range Signal Measurements. IEEE Trans. Instrum. Meas.

[9] Song Y., Zhang K., Hao Q., Rolland P. (2012). Modeling and Characterization of the Electrostatic Coupling Intra-Body Communication Based on Mach-Zehnder Electro-Optical Modulation. Opt. Express.

[10] Song Y., Hao Q., Zhang K., Wang M., Chu Y., Kang B. (2011). The Simulation Method of the Galvanic Coupling Intra-Body Communication with Different Signal Transmission Paths. IEEE Trans. Instrum. Meas.

[11] Wegmueller M.S., Hediger M., Kaufmann T., Buergin F., Fichtner W. Wireless Implant Communications for Biomedical Monitoring Sensor Network.

[12] Boyd R.W. (2008). Nonlinear Optics.

[13] Lu G.N., Sou G. A CMOS Op Amp Using a Regulated-Cascode Transimpedance Building Block for High-Gain, Low-Voltage Achievement.

[14] Callejón M.A., Naranjo-Hernandez D., Reina-Tosina J., Roa L.M. (2012). Distributed Circuit Modeling of Galvanic and Capacitive Coupling for Intrabody Communication. IEEE Trans. Biomed. Eng.

[15] Cho N., Yoo J., Song S.-J., Lee J., Jeon S., Yoo H.-J. (2007). The Human Body Characteristics as a Signal Transmission Medium for Intrabody Communication. IEEE Trans. Microw. Theory Tech.

